# DVS Benchmark Datasets for Object Tracking, Action Recognition, and Object Recognition

**DOI:** 10.3389/fnins.2016.00405

**Published:** 2016-08-31

**Authors:** Yuhuang Hu, Hongjie Liu, Michael Pfeiffer, Tobi Delbruck

**Affiliations:** Institute of Neuroinformatics, University of Zurich and ETH ZurichZurich, Switzerland

**Keywords:** neuromorphic, event-based vision, AER, benchmarks, DVS, action recognition, object tracking, object recognition

## 1. Introduction

Benchmarks have played a vital role in the advancement of visual object recognition and other fields of computer vision (LeCun et al., 1998; Deng et al., 2009;). The challenges posed by these standard datasets have helped identify and overcome the shortcomings of existing approaches, and have led to great advances of the state of the art. Even the recent massive increase of interest in deep learning methods can be attributed to their success in difficult benchmarks such as ImageNet (Krizhevsky et al., 2012; LeCun et al., 2015). Neuromorphic vision uses silicon retina sensors such as the dynamic vision sensor (DVS; Lichtsteiner et al., 2008). These sensors and their DAVIS (Dynamic and Active-pixel Vision Sensor) and ATIS (Asynchronous Time-based Image Sensor) derivatives (Brandli et al., 2014; Posch et al., 2014) are inspired by biological vision by generating streams of asynchronous events indicating local log-intensity brightness changes. They thereby greatly reduce the amount of data to be processed, and their dynamic nature makes them a good fit for domains such as optical flow, object tracking, action recognition, or dynamic scene understanding. Compared to classical computer vision, neuromorphic vision is a younger and much smaller field of research, and lacks benchmarks, which impedes the progress of the field. To address this we introduce the largest event-based vision benchmark dataset published to date, hoping to satisfy a growing demand and stimulate challenges for the community. In particular, the availability of such benchmarks should help the development of algorithms processing event-based vision input, allowing a direct fair comparison of different approaches. We have explicitly chosen mostly dynamic vision tasks such as action recognition or tracking, which could benefit from the strengths of neuromorphic vision sensors, although algorithms that exploit these features are largely missing.

A major reason for the lack of benchmarks is that currently neuromorphic vision sensors are only available as R&D prototypes. Nonetheless, there are several datasets already available; see Tan et al. (2015) for an informative review. Unlabeled DVS data was made available around 2007 in the jAER project^1^ and was used for development of spike timing-based unsupervised feature learning e.g., in Bichler et al. (2012). The first labeled and published event-based neuromorphic vision sensor benchmarks were created from the MNIST digit recognition dataset by jiggling the image on the screen (see Serrano-Gotarredona and Linares-Barranco, 2015 for an informative history) and later to reduce frame artifacts by jiggling the camera view with a pan-tilt unit (Orchard et al., 2015). These datasets automated the scene movement necessary to generate DVS output from the static images, and will be an important step forward for evaluating neuromorphic object recognition systems such as spiking deep networks (Pérez-Carrasco et al., 2013; O'Connor et al., 2013; Cao et al., 2014; Diehl et al., 2015), which so far have been tested mostly on static image datasets converted into Poisson spike trains. But static image recognition is not the ideal use case for event-based vision sensors that are designed for dynamic scenes. Recently several additional DVS datasets were made available in the Frontiers research topic “Benchmarks and Challenges for Neuromorphic Engineering”^2^; in particular for navigation using multiple sensor modalities (Barranco et al., 2016) and for developing and benchmarking DVS and DAVIS optical flow methods (Rueckauer and Delbruck, 2016).

This data report summarizes a new benchmark dataset in which we converted established visual video benchmarks for object tracking, action recognition and object recognition into spiking neuromorphic datasets, recorded with the DVS output (Lichtsteiner et al., 2008) of a DAVIS camera (Berner et al., 2013; Brandli et al., 2014). This report presents our approach for sensor calibration and capture of frame-based videos into neuromorphic vision datasets with minimal human intervention. We converted four widely used dynamic datasets: the VOT Challenge 2015 Dataset (Kristan et al., 2016), TrackingDataset^3^, the UCF-50 Action Recognition Dataset (Reddy and Shah, 2012), and the Caltech-256 Object Category Dataset (Griffin et al., 2006). We conclude with statistics and summaries of the datasets.

## 2. Materials and methods

The DVS data are generated by displaying existing benchmark videos on a monitor, and recording with a stationary DAViS240C vision sensor under controlled lighting conditions. Because of the dynamic nature of the displayed video, the sensor will generate events for local brightness changes. Because the original datasets are frame based, we characterized the artifacts produced by the stroboscopic video sequence presentations and monitor refresh rate.

### 2.1. Benchmark recording setup

**Figure 1:**
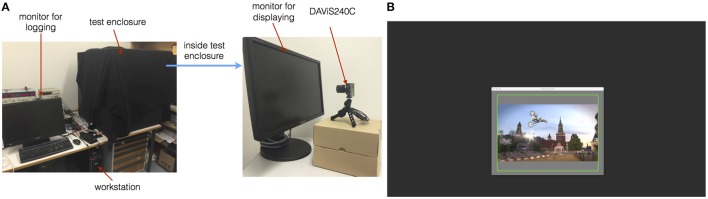
**(A)** Experiment environment setup used in this project. **(B)** Monitor display for sequences.

Figure 1A illustrates the setup for generating recordings with neuromorphic vision sensors, thereby converting the existing benchmark datasets. The setup consists of a test enclosure for controlling the lighting conditions. Inside the enclosure is a consumer-grade TFT LCD monitor (Samsung SyncMaster 2343BW) with a refresh rate of 60 Hz and the native resolution of 2048 × 1152, that displays the original video sequences and is the only light source. The monitor was set to its highest brightness and contrast setting. The display is recorded with a DAViS240C neuromorphic vision sensor^4^, recording events at a resolution of 240 × 180; (Berner et al., 2013; Brandli et al., 2014). The sensor uses default bias settings, and recording of DAVIS APS (Active Pixel Sensor) frames, i.e., frame-based intensity read-outs at regular sampling intervals, is deactivated to reduce the dataset sizes. An Ubuntu 14.04 LTS workstation outside of the enclosure controls the video display of the dataset, with a second LCD display for controlling and monitoring the recording. Recording of AER (Address-Event Representation) events, the most commonly used representation of event data, is done with the jAER software^5^. We also developed a Python package called SpikeFuel^6^, which is released together with the datasets and is used for displaying and scheduling video sequences, as well as post-processing. SpikeFuel displays frames using OpenCV and controls jAER using local UDP datagrams using jAER's Remote Control protocol.

### 2.2. Recording procedure

For each dataset the position of the DAViS240C is adjusted so its field of view covers the region of interest on the monitor, which is a 4:3 aspect ratio window in the center of the screen, surrounded by gray background of 50% intensity. This alignment is achieved by displaying a flashing green rectangle (Figure 1B). Also, the video sequence is rescaled to fit the size of the field of view of the DAViS240C. To make sure that every frame of the sequence is displayed at least once during the monitor's refreshing period, the video is played at a frame-rate equal or lower than the monitor's refresh rate, in our case at 30 Hz, which is also the original frame rate of the videos. In principle, display at higher rates is possible, but the interplay between GPU rendering and monitor refreshing can become unreliable. The recording of each video starts with an adaptation period of 2 s, in which only the initial frame of the video sequence is displayed. This eliminates unwanted event bursts due to flashing a video on a background screen. Before the playback of the video is started, the jAER timestamps are reset to 0, then the recording is started. At the end of a sequence the recording is saved, while the last frame of the sequence is displayed for 2 s. In post-processing the transition from first to second video frame is detected by the initial burst of DVS activity. For tracking datasets, the bounding box coordinates are transformed to DAViS240C coordinates and supplied with the data along with the corresponding DAViS240C timestamp.

## 3. Results

We converted four benchmark sets of videos, for tracking, action recognition, and object classification. All videos had a preset display frame rate of 30 fps (frames per second) except for the Caltech-256 which used 10 fps. These datasets are available at http://sensors.ini.uzh.ch/databases.html. This website provides instructions on how to access the datasets, specific instructions on how to display the data using jAER, and presents screenshots and demo videos of the datasets. Furthermore, the website contains instructions on how to use the SpikeFuel tool for generating new datasets, including example code and extra notes. The characteristics of the four datasets are summarized in Table 1, and they are described in detail below.

**Table 1:** **Characteristics of the four provided DVS benchmark datasets**.

**Name**	**Domain**	**Nr. Recordings**	**Avg. Length/Recording (s)**	**Max. FR (keps)**	**Avg. FR (keps)**
VOT Challenge 2015	Tracking	60	12.25	383.63	251.85
TrackingDataset	Tracking	67	20.70	342.07	197.77
UCF-50	Action Recognition	6676	6.80	238.11	162.62
Caltech-256	Object Recognition	30607	1.01	N/A	110.57

*For each dataset the number of available sequence recordings, the average length of the recordings, the maximum firing rate (FR) and the average firing rate in keps (kilo events per second) are specified*.

### 3.1. VOT challenge 2015 dataset DVS recordings

**Figure 2:**
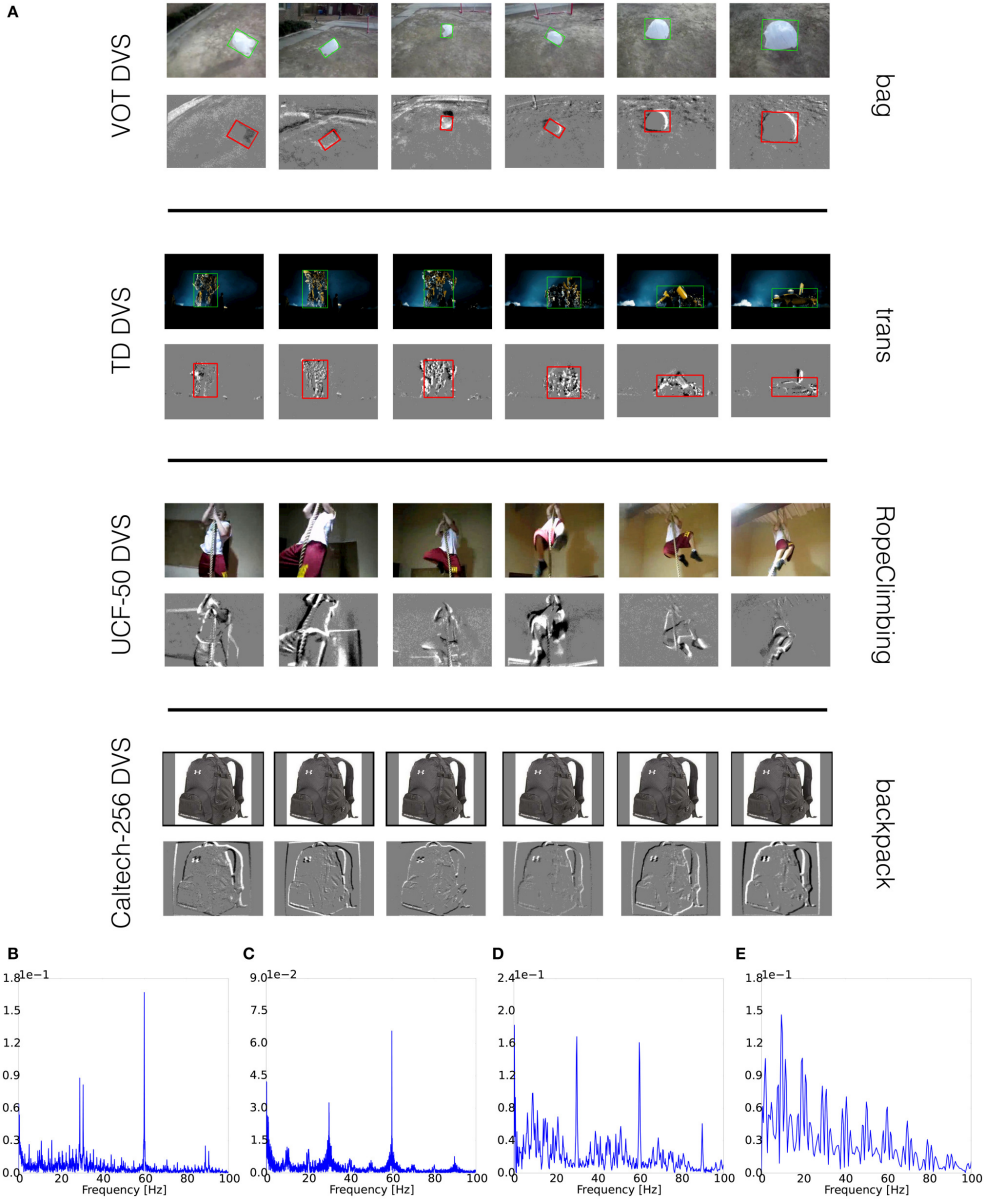
**(A)** Screenshots of Datasets; **(B)** Amplitude Spectra of VOT Dataset DVS recording; **(C)** Amplitude Spectra of TrackingDataset DVS recording; **(D)** Amplitude Spectra of UCF-50 DVS recording; **(E)** Amplitude Spectra of Caltech-256 DVS recording.

The *VOT Challenge 2015 Dataset* consists of 60 single-object tracking sequences, many with challenging moving background (examples in Figure 2A first row). The average number of frames is 358. The first row of Figure 2A shows an example from DVS recordings. The bounding boxes are post-computed according to the ground truth in the original sequence. The amplitude spectrum of one representative sequence (bolt2) in the dataset (Figure 2B) shows there are event bursts around both 30 (preset FPS) and 60 Hz (monitor refresh rate). The spectrum is generated using the same method as in supplementary materials of Serrano-Gotarredona and Linares-Barranco (2015), where also methods are described to potentially remove artifacts. Since other post-processing techniques could be used, we have decided to provide the original, unprocessed datasets.

### 3.2. Tracking dataset DVS recordings

The *TrackingDataset* has 77 single-object tracking sequences (examples in Figure 2A second row). The average number of frames per sequence is 605. Due to memory constraints for the smooth display of very long sequences, the category “Kalal” was excluded. The second row of Figure 2A gives a closer look of the acquired recordings. The original and transformed bounding boxes of the sequence trans are displayed. Fourier analysis of the TrackingDataset recordings shows similar structure as for the VOT Dataset, indicating event bursts at 30 and 60 Hz (Figure 2C).

### 3.3. UCF-50 action recognition dataset DVS recordings

The *UCF-50 Action Recognition Dataset* consists of 6676 videos in 50 action classes (screenshots in Figure 2A third row). The average length of videos is 6.64 s. The third row of Figure 2A shows recordings for the RopeClimbing sequence, which is representative of samples with static background and reasonable lighting conditions, so that details of actions are dominant. The Fourier analysis of one recording is presented in Figure 2D. It shows similar structure as in the previous two datasets.

### 3.4. Caltech-256 object recognition dataset DVS recordings

The *Caltech-256 Object Recognition Dataset* (Griffin et al., 2006) has 30,607 still images that are categorized in 257 classes (example in Figure 2A, fourth row). Each class has 119 images on average. For each image, 10 small saccades presented at 10 fps were used to introduce movement. These saccades are drawn from a uniform distribution in the range ±3 pixels for both horizontal and vertical axes. All remaining experiment procedures are the same as for other datasets. The spectral analysis displays bursts at 10 Hz and harmonics, as in previous datasets (Figure 2E).

## 4. Discussion

There are a total of 37,410 recordings, representing the largest neuromorphic vision datasets for these domains of machine vision. A software pipeline for capturing frame-based visual recognition benchmarks with neuromorphic cameras was developed. Datasets are delivered in both HDF5 and AEDAT-2.0 AER raw data format (so far there is no HDF5 parser in jAER). We hope that these recordings can boost the development of event-based learning in visual tasks.

In some tracking sequences, the target objects are still, or cannot be differentiated from the background (e.g., rabbit running on snowy ground). And in some action recognition sequences, the background is rapidly moving. These factors that are introduced by original datasets show that a stationary DVS is not always sufficient for solving dynamic vision applications.

The 30 Hz sample rate of the original recordings aliases information above 15 Hz in the original scene. The artifacts in the DVS output that are caused by the frames in the original datasets show that it is necessary to use neuromorphic sensors for collection of new frame-free datasets that will take full advantage of the precise timing of such sensors, which may be crucial for optical flow computation or event-based stereo (Rogister et al., 2012; Rueckauer and Delbruck, 2016). However, the datasets presented here provide a valuable basis for the development of higher-level algorithms processing and recognizing event-based spatio-temporal patterns, such as in tracking and action recognition applications. By providing common benchmarks for these areas we expect a more solid comparison of the (few) existing approaches, and to aid the development of novel algorithmic ideas.

## Author contributions

YH performed the recordings. YH, HL, MP, and TD designed the experimental setup and drafted the manuscript.

## Funding

This research is supported by the European Commission project VISUALISE (FP7-ICT-600954), SeeBetter (FP7-ICT-270324), and the Samsung Advanced Institute of Technology.

### Conflict of interest statement

TD is a minority shareholder and CSO of inilabs GmbH. The other authors declare that the research was conducted in the absence of any commercial or financial relationships that could be construed as a potential conflict of interest.

## Footnotes

^1^Available at https://sourceforge.net/p/jaer/wiki/AER%20data/

^2^Available at http://journal.frontiersin.org/researchtopic/3448/benchmarks-and-challenges-for-neuromorphic-engineering

^3^Available at http://cmp.felk.cvut.cz/~vojirtom/dataset/

^4^http://inilabs.com/products/dynamic-and-active-pixel-vision-sensor/

^5^Available at http://jaerproject.org

^6^Available at https://github.com/duguyue100/spikefuel
